# Inflammatory Stewardship—A Perspective for Active Management of Acute Inflammation

**DOI:** 10.1096/fj.202600607R

**Published:** 2026-06-24

**Authors:** Lennart Finn Herrlich, Hubert Schrezenmeier, David Alexander Christian Messerer

**Affiliations:** ^1^ Institute of Transfusion Medicine and Immunogenetics Ulm German Red Cross Blood Transfusion Service Baden‐Württemberg‐Hessen and University Hospital Ulm Ulm Germany; ^2^ Institute of Transfusion Medicine University Hospital Ulm Ulm Germany

## Abstract

Major surgery and traumatic injury are potent triggers of systemic inflammation. While appropriately regulated inflammation supports host defense and tissue repair, dysregulated and time‐dependent inflammatory trajectories contribute to postoperative and post‐traumatic complications, organ dysfunction, prolonged disability, and mortality. Translation of mechanistic insight into effective bedside strategies has been limited by marked inter‐individual heterogeneity, rapid phase shifts in the inflammatory response, and the absence of operational workflows that measure inflammation with sufficient biological and temporal resolution to guide patient‐specific decisions. This perspective introduces ‘inflammatory stewardship’ as a precision‐medicine paradigm for proactive management of acute inflammation in perioperative and critical care settings. Inflammatory stewardship integrates two core components: inflammatory staging and biomarker‐guided immunomodulation. Inflammatory staging uses serial, multidimensional monitoring to characterize trajectories and to determine whether a patient's current inflammatory status lies within an individualized target range. Monitoring may combine routinely available clinical parameters with more specific immune phenotyping, functional assays, and molecular signatures, provided that assays are standardized, clinically interpretable, and available within actionable turnaround times. Biomarker‐guided immunomodulation then links off‐target trajectories to patient‐specific escalation or de‐escalation strategies, supported by predefined safety criteria and interdisciplinary governance. This perspective outlines key implementation requirements and a research agenda to establish reference trajectories, validate actionable endotypes, and embed biomarkers into adaptive interventional study designs. Inflammatory stewardship offers a testable roadmap to operationalize personalized inflammation management after surgery and trauma, aiming to enable earlier detection of maladaptive trajectories and more effective, patient‐tailored immunomodulatory care in intensive care.

## Graphical abstract

Inflammatory stewardship frames inflammation as a manageable clinical domain requiring explicit strategy, governance, and feedback. It aligns diagnostics, risk stratification, biomarker interpretation, and immunomodulatory decisions across perioperative and critical‐care teams. Strategically, the concept prioritizes high‐risk patients, defines phase‐specific target ranges, standardizes monitor–act–reassess loops, and links deviation from expected trajectories to predefined escalation or de‐escalation. Clinically, it aims to improve trauma and perioperative care by enabling earlier recognition of maladaptive trajectories, safer individualized intervention, and more consistent recovery‐oriented management at scale.

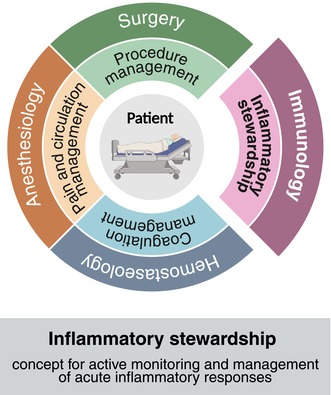

**Abbreviations**
Act: activator
APCs: antigen‐presenting cells
ARDS: acute respiratory distress syndrome
C3: complement component 3
C4: complement component 4
C5: complement component 5
C5a: complement component 5a
CABG: coronary artery bypass grafting
CAR‐T: chimeric antigen receptor T cell
CBC: complete blood count
CRP: C‐reactive protein
DAMP(s): damage‐associated molecular pattern(s)
DCO: damage‐control orthopedics
DCS: damage‐control surgery
ECMO: extracorporeal membrane oxygenation
ELISA: enzyme‐linked immunosorbent assay
EMSA: electrophoretic mobility shift assay
ERAS: Enhanced Recovery After Surgery
ESR: erythrocyte sedimentation rate
ETC: early total care
FiO_2_
: fraction of inspired oxygen
GM‐CSF: granulocyte‐macrophage colony‐stimulating factor
HIS: hospital information system
HLA‐DR: human leukocyte antigen‐DR
HMGB1: high mobility group box 1
ICU: intensive care unit
IFN‐γ: interferon gamma
IL: interleukin
IL‐1β: interleukin‐1 beta
IL‐2: interleukin‐2
IL‐6: interleukin‐6
IL‐7: interleukin‐7
INR: international normalized ratio
IVD: in vitro diagnostic
IVIG: intravenous immunoglobulin
LIS: laboratory information system
LPS: lipopolysaccharide
MI: myocardial infarction
MRI: magnetic resonance imaging
NETosis: neutrophil extracellular trap formation
NF‐κB: nuclear factor kappa B
NLRP3: NLR family pyrin domain‐containing 3
PAMP(s): pathogen‐associated molecular pattern(s)
PaO_2_
: arterial partial pressure of oxygen
PaO_2_/FiO_2_
: ratio of arterial partial pressure of oxygen to fraction of inspired oxygen
PBMC(s): peripheral blood mononuclear cells
PCT: procalcitonin
PD‐1: programmed cell death protein 1
PD‐L1: programmed death‐ligand 1
PV: plasma viscosity
RCT: randomized controlled trial
RNA‐seq: RNA sequencing
RWE: real‐world evidence
SIRS: systemic inflammatory response syndrome
SOFA: sequential organ failure assessment
TLR: Toll‐like receptor
TNF‐α: tumor necrosis factor alpha
Treg(s): regulatory T cell(s)
WBC: white blood cell count
WES: whole exome sequencing
WGS: whole genome sequencing

## 1 Introduction

Major surgery and traumatic injury are high‐burden, high‐heterogeneity events. Globally, more than 300 million surgical procedures are performed each year, with an estimated 4.2 million deaths occurring in the 30 days following surgery [1]. Likewise, traumatic injuries remain among the leading causes of death and disability, disproportionately affecting younger populations and resulting in profound personal, societal, and economic consequences [2].

Advances in modern medicine have enabled increasingly complex surgical interventions, extending treatment possibilities far beyond previous limits. Simultaneously, these procedures are often performed in an aging and frail population with a high burden of comorbidities and therefore an increased vulnerability to complications [3]. Across the overall surgical population, severe adverse outcomes are uncommon [4]. However, clinically relevant dysregulated inflammatory trajectories and prolonged critical‐care use concentrate in identifiable high‐risk subsets (e.g., frailty, comorbidity, major abdominal surgery, hemorrhagic shock, severe tissue injury) and are associated with organ dysfunction and mortality [5, 6]. Therefore, both the scope and the stakes of personalized, trajectory‐aware management are greater than ever.

Major surgical procedures and traumatic injuries originate from different causes. However, both are potent triggers of systemic inflammation [7, 8]. Both settings provide well‐defined ‘time‐zero’ models for acute inflammation. The stewardship principles proposed are intended to be transferable to other acute critical‐illness syndromes (e.g., sepsis), where staging and conservative, protocolized modulation are equally needed [9, 10]. Tissue damage leads to the release of damage‐associated molecular patterns (DAMPs) and exposure to pathogen‐associated molecular patterns (PAMPs) through a disruption of physical barriers against microbial invasion [7, 11]. These danger signals activate cascades of local and systemic inflammatory pathways [12, 13], orchestrating the inflammatory response of the host. While this process is essential for initiating tissue repair and preventing or limiting pathogen translocation across compromised barriers [11, 14], in some patients, the acute inflammatory response becomes dysregulated in magnitude, timing, and compartmentalization [13]. Such dysregulation contributes to complications, impairs recovery, promotes progression of chronic diseases and/or prompts organ dysfunction, and ultimately increases long‐term disability or even mortality [6, 8, 15, 16, 17, 18]. Critically, clinically similar syndromes in intensive care medicine may reflect distinct inflammatory states, which is the biological premise for staging inflammation rather than treating inflammation as a single therapeutic target.

Altogether, the clinical umbrella term inflammation may refer to biologically highly distinct states. For example, early after major surgery, inflammation may refer to the appropriate and even necessary processes that facilitate host defense and initiate wound healing and tissue repair. By contrast, for a patient with myocardial ischemia, cardiogenic shock, secondary infection, and organ dysfunction, the same term can simultaneously encompass sterile DAMP‐driven inflammation, infection‐related PAMP signaling, endothelial activation, and evolving immunosuppression. In a third pattern, inflammation may refer to persistent low‐grade vascular processes in patients with high cardiovascular risk, which may be less explosive in the acute setting but remain clinically relevant over longer time scales. These examples illustrate that sterile tissue injury, microbial danger signaling, endothelial dysfunction, organ injury, and immune suppression may coexist and evolve asynchronously within the same patient. Accordingly, inflammation cannot be interpreted as a single scalar variable, because even widely used markers such as CRP require temporal, clinical, and mechanistic context [7, 8, 11, 12, 13, 19, 20].

Consequently, addressing inflammation clinically remains challenging, as this complex heterogeneity complicates therapeutic decision‐making and limits the effectiveness of one‐size‐fits‐all immunomodulatory approaches. Therefore, this perspective advocates for a precision‐medicine approach to proactive monitoring and modulation of acute inflammatory responses in the context of major surgery and trauma. It outlines the major challenges of such a strategy and discusses reasons why the management of acute inflammation currently exhibits translational gaps, comparable to those previously encountered in other medical disciplines. In addition, this perspective hypothesizes about potential future developments that could accelerate clinical progress in the field of inflammation management. Finally, it proposes an ‘inflammatory stewardship’ framework linking individualized inflammatory staging to biomarker‐guided escalation and de‐escalation strategies.

## 2 Main Body

### 2.1 Making a Case for Inflammatory Stewardship

Despite expectable perioperative immune activation, there is currently no validated, patient‐tailored, biomarker‐guided framework to routinely monitor and act on acute inflammation in major surgery or trauma. While there are many established biomarkers that are applied in the clinic to monitor inflammation, these markers exhibit important limitations as discussed later and rarely capture the dynamic, multidimensional nature of inflammatory activity. Yet, such a comprehensive assessment is a prerequisite for personalized therapeutic decision‐making and for matching immunomodulatory strategies to the right patient at the right time.

In contrast, biomarker‐guided care is significantly more advanced in several other domains. For example, in hemostasis and coagulation management (hemostaseology), numerous laboratory parameters such as platelet count, prothrombin time/INR, and fibrinogen are routinely applied to orchestrate targeted interventions within patient blood management protocols and perioperative bleeding‐management algorithms [21, 22]. Likewise, preexisting comorbidities are systematically incorporated into preoperative evaluation, risk stratification, and perioperative planning [23]. In the future, genome‐wide data, ranging from clinically actionable pharmacogenomic variants to polygenic risk scores, may be incorporated to personalize perioperative decision‐making, contingent on robust prospective evidence of clinical benefit [24, 25].

Such biomarker‐guided management workflows enable precise, individualized interventions and are embedded in routine practice across several medical domains [21, 22, 23, 26, 27]. By contrast, the management of acute inflammatory responses remains comparatively underdeveloped. Because clinically relevant dysregulated trajectories occur in a substantial high‐risk subset after major surgery and trauma [7, 8, 12], the absence of a structured monitor–act–reassess framework, especially in deteriorating patients requiring intensive care, is striking.

The need for such a framework is underscored by unstratified cardiac‐surgery corticosteroid trials, which did not show outcome benefit sufficient to support routine perioperative use [15, 16], a result consistent with the biological heterogeneity of perioperative inflammation across patients, procedures, and time points [13]. These observations support a shift toward personalized inflammation management, linking biomarker‐defined endotypes and time‐dependent trajectories to individualized timing, dosing, and escalation/de‐escalation algorithms.

**FIGURE 1:**
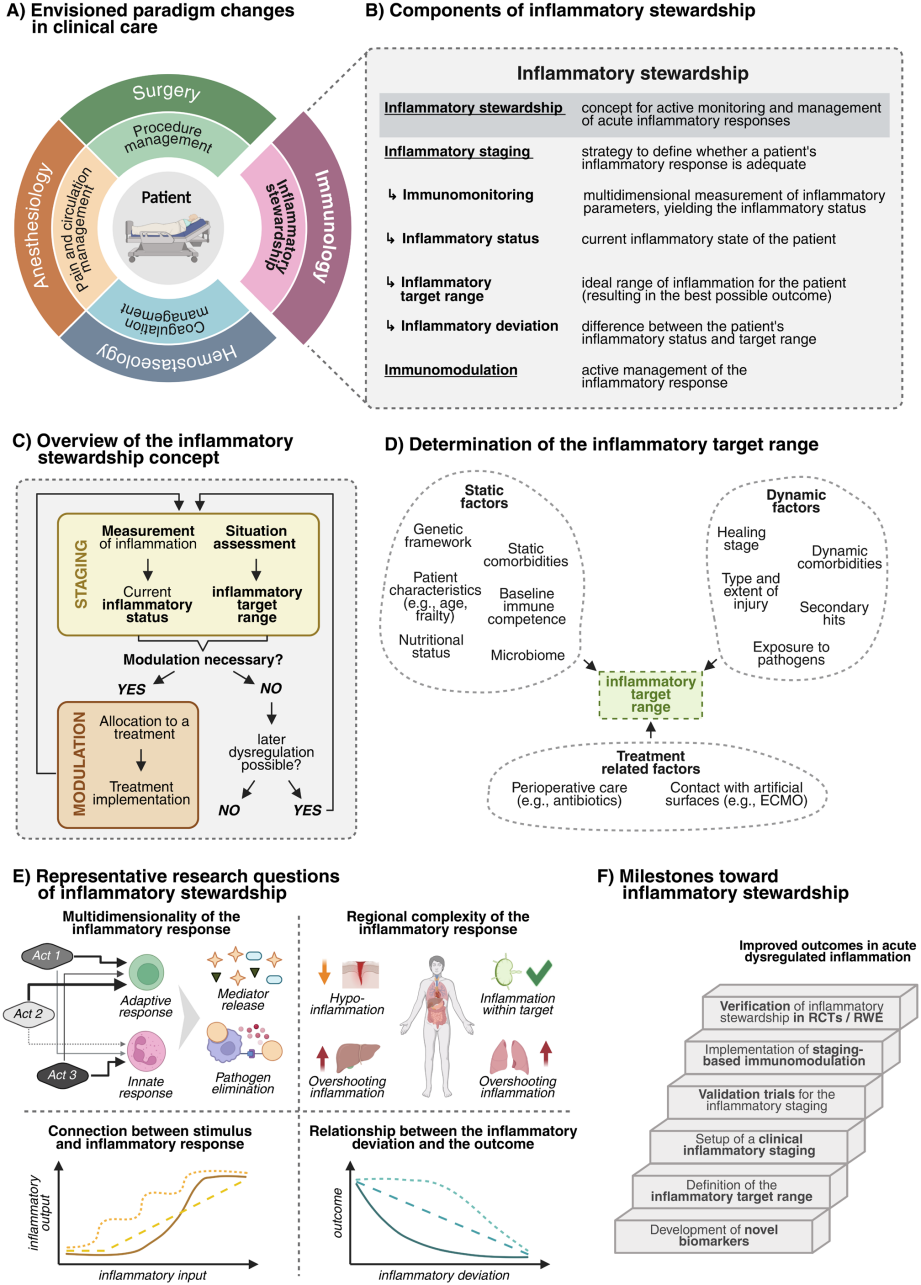
The vision of inflammatory stewardship and an overview of its components. (A) The envisioned paradigm change in the management of acute inflammation in patients with major surgery and trauma is the proactive holistic incorporation of immunological aspects. (B) A complete inflammatory stewardship contains multiple components and related terminology. (C) The concept of inflammatory stewardship in a schematic flow chart. (D) Factors influencing the inflammatory target range. The range depends on static, dynamic, and treatment‐related factors. (E) Toward true inflammatory stewardship, multiple knowledge gaps and theoretical uncertainties must be addressed: Which components of the multidimensional immune response should be measured or targeted? Are there spatiotemporal differences in the dysregulation of acute inflammatory responses? How are inflammatory inputs/stimuli connected to the inflammatory output? What is the relationship between a deviation from the inflammatory target range and the patient's outcome? (F) Major steps and challenges leading toward inflammatory stewardship. Act, activator; ECMO, extracorporeal membrane oxygenation; RCT, randomized controlled trial; RWE, real‐world evidence.

Taken together, acute inflammation after major surgery and trauma requires a structured precision‐medicine framework (Figure 1). We use the term inflammatory stewardship for a conservative monitor–act–reassess approach with two components: inflammatory staging, which determines whether the patient's current trajectory lies within an individualized target range, and biomarker‐guided immunomodulation, which corrects off‐target trajectories through predefined escalation or de‐escalation.

**FIGURE 2:**
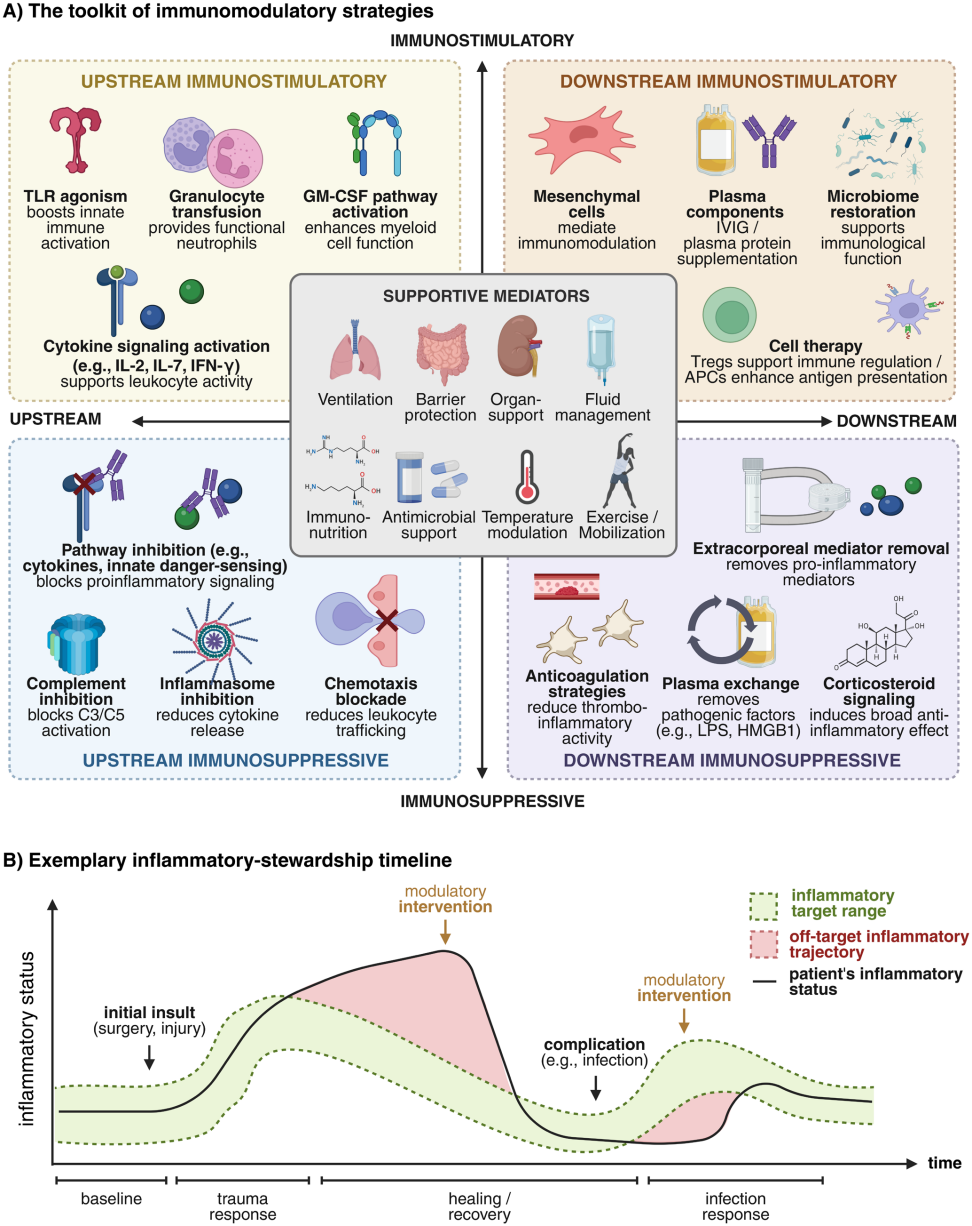
Overview of inflammatory stewardship and associated immunomodulation. (A) Conceptual four‐quadrant landscape of immunomodulatory interventions, positioned according to their predominant biological level of action, from upstream mechanistic targeting to downstream consequence modulation, and their dominant immune direction, from immunostimulation to immunosuppression. Direct immune‐targeted interventions are placed within the quadrants, whereas context‐dependent supportive modulators with less specific or variable positioning are shown at the axis intersection. The examples shown are illustrative and should not be read as therapeutic recommendations. Placements reflect the predominant mechanism and typical point of action only. The stimulatory–suppressive and upstream–downstream axes represent continua rather than discrete categories. Many interventions are pleiotropic and may act in a net immunomodulatory (bidirectional) manner. This applies particularly to cellular therapies. Antigen‐presenting‐cell‐based approaches may enhance immune activation, whereas regulatory T‐cell‐based approaches are primarily immunoregulatory or immunosuppressive. Their shared placement in the schematic therefore denotes the broader category of cellular immunomodulation, not a uniform immunostimulatory effect. The actual effect is context dependent. It varies with the individual patient and immune state, the inflammatory trigger (e.g., surgery, trauma, or infection), and the dose and timing of the intervention. (B) Exemplary inflammatory timeline illustrating how inflammatory staging may detect deviation of the patient's inflammatory status from the inflammatory target range and thereby enable timely adaptation of inflammatory management. APCs, antigen‐presenting cells; C3, complement component 3; C5, complement component 5; GM‐CSF, granulocyte‐macrophage colony‐stimulating factor; HMGB1, high mobility group box 1; IFN‐γ, interferon gamma; IL‐2, interleukin‐2; IL‐7, interleukin‐7; IVIG, intravenous immunoglobulin; LPS, lipopolysaccharide; TLR, Toll‐like receptor; Tregs, regulatory T cells.

The first critical component of this personalized inflammation‐management concept is to determine whether the patient's current inflammatory status and trajectory fall within an individualized target range (Figure 2). This inflammatory staging process consists of repetitive measurements of the patient's current inflammatory state, which is then quantified as the patient's ‘inflammatory status’. This status is then compared to a phase‐specific, risk‐adjusted reference trajectory band (‘inflammatory target range’) that is empirically associated with predefined favorable outcomes (e.g., no new organ dysfunction, no secondary infection, timely recovery). For early implementation, such target ranges can be derived from large observational cohorts and trial biobanks as reference trajectories and progressively refined toward individualization, with re‐calibration after trigger events (e.g., reoperation, major bleeding, new infection) [6, 28, 29, 30, 31, 32].

Inflammatory stewardship is inspired by established ICU stewardship frameworks (e.g., antimicrobial stewardship and patient blood management), but the analogy is imperfect: inflammatory signals are multidimensional and causal links to interventions are often indirect. Therefore, stewardship must be conservative, safety‐centric, and trial‐informed, emphasizing avoidance of both over‐ and under‐treatment with tight feedback loops [21, 22, 33]. Beyond improving clinical effectiveness, such frameworks support safer, higher‐value care by standardizing decision‐making and reducing unwarranted practice variation [27, 34, 35]. As in antimicrobial stewardship and coagulation management, effective inflammatory stewardship requires accurate diagnostics and risk stratification, evidence‐based intervention choice, appropriate timing and dose/intensity, continuous monitoring with feedback for de‐escalation or escalation, and tight interdisciplinary teamwork (Figure 1).

### 2.2 Exploring Key Concepts of Inflammatory Stewardship

#### 2.2.1 Capturing Individual Inflammatory Responses is Key for the Success of Immunomodulation

The first component of inflammatory staging is the measurement of the patient's current inflammatory status. To this end, multiple established and novel methods exist. A comprehensive catalog of candidate biomarkers across indications is beyond the scope of this perspective. Selected established and emerging measurements are summarized in Table 1. Notably, important research gaps remain in the pathophysiology of acute inflammation, such as the structure of activation networks and the quantitative relationships between inflammatory inputs and outputs (e.g., linear, logarithmic, or threshold effects (Figure 1)).

**TABLE 1:** Selected clinical, laboratory, cellular, and molecular measurements for assessing acute inflammation.

Category	Measurement	Method	Sample type	Established use/availability	Notes
Clinical markers and measurements	Scoring systems (e.g., systemic inflammatory response syndrome (SIRS), sequential organ failure assessment (SOFA))	Composite clinical scores	Bedside assessment	+++	Widely used in the ICU setting, low specificity
	Imaging methods	MRI, ultrasound	Patient/organ‐system imaging	+++	Identify organ inflammation, structural damage, and abscesses
	Markers of organ function (heart rate, blood pressure, PaO_2_/FiO_2_)	Bedside monitoring, hemodynamic monitoring, blood gas analysis	Multiple	+++	Identify organ dysfunction
	Erythrocyte sedimentation rate (ESR)/plasma viscosity (PV)	ESR‐assay/PV‐assay	Whole blood/plasma	++	Low specificity
Established markers of the immune response	Acute‐phase proteins (C‐reactive protein (CRP), serum amyloid A, haptoglobin, fibrinogen)	Enzyme‐linked immunosorbent assay (ELISA), nephelometry, immunoturbidimetry	Plasma/serum	+++	Highly common markers for assessing systemic inflammation
	Cytokines (e.g., IL‐6, TNF‐α)	ELISA, multiplex bead assays	Plasma/serum	++	Reflect immunological processes
	Complement components and activation products (e.g., C3, C4, C5a)	ELISA, nephelometry, functional assays	Plasma/serum	++	Involved in inflammatory processes
	Coagulation/contact‐pathway markers (e.g., kallikrein, thrombin, tissue factor)	Activity assays, ELISA	Plasma	++	Reflect link between inflammation and coagulation
	Procalcitonin (PCT)	Immunoassay (chemiluminescence, ELISA)	Plasma/serum	+++	More infection‐oriented than CRP in some settings, may support antibiotic stewardship when interpreted in clinical context.
	White blood cell count (WBC)	Automated hematology analyzers	Whole blood	+++	Routine and inexpensive
Novel/experimental markers of the immune response	Functional assays (e.g., NETosis assays)	Multiple (e.g., fluorescence microscopy)	Whole blood	+	Explore cellular functions
	Transcription factor activities (e.g., NF‐κB)	Reporter assays, EMSA, phospho‐flow	PBMCs, leukocytes	+	Provide mechanistic insights into signaling processes
	Transcriptomic signatures	RNA‐seq, microarrays	Whole blood, leukocytes	+	Captures early transcriptional alterations
	Expression of surface markers (e.g., HLA‐DR on monocytes, PD‐1/PD‐L1 on leukocytes)	Flow cytometry	Whole blood, PBMCs, leukocytes	+	Captures alterations on a cellular level
	Genetics (germline/somatic) and epigenetics	Whole genome sequencing (WGS), whole exome sequencing (WES), methylation microarrays	PBMCs, leukocytes, tissue biopsies	+	Detects genetic susceptibility (germline), acquired somatic variants, and epigenetic changes

*Note:* The table summarizes established and emerging approaches that may inform inflammatory staging in perioperative and critical‐care settings. Measurements are grouped into clinical markers and measurements, established immune‐response markers, and novel or experimental immune‐response markers. Availability is graded semi‐quantitatively as high (+++), moderate (++), or limited/experimental (+). Availability grading is approximate and context‐dependent, not an evidence‐grade assessment. The list is illustrative rather than exhaustive and emphasizes clinical implementability, biological proximity to inflammatory processes, and potential relevance for inflammatory stewardship.

Abbreviations: C3, complement component 3; C4, complement component 4; C5a, complement component 5a; CRP, C‐reactive protein; ELISA, enzyme‐linked immunosorbent assay; EMSA, electrophoretic mobility shift assay; ESR, erythrocyte sedimentation rate; FiO_2_, fraction of inspired oxygen; HLA‐DR, human leukocyte antigen‐DR; ICU, intensive care unit; IL‐6, interleukin‐6; MRI, magnetic resonance imaging; NETosis, neutrophil extracellular trap formation; NF‐κB, nuclear factor kappa B; PaO_2_, arterial partial pressure of oxygen; PaO_2_/FiO_2_, ratio of arterial partial pressure of oxygen to fraction of inspired oxygen; PBMCs, peripheral blood mononuclear cells; PCT, procalcitonin; PD‐1, programmed cell death protein 1; PD‐L1, programmed death‐ligand 1; PV, plasma viscosity; RNA‐seq, RNA sequencing; SIRS, systemic inflammatory response syndrome; SOFA, sequential organ failure assessment; TNF‐α, tumor necrosis factor alpha; WBC, white blood cell count; WES, whole exome sequencing; WGS, whole genome sequencing.

In current practice, the quantification of inflammation relies on a combination of immunological biomarkers (e.g., cytokines), markers of organ dysfunction (e.g., creatinine, or composite scores such as SOFA/SIRS), and clinical parameters (e.g., body temperature) [7, 8, 12]. While immune biomarkers can reflect upstream immune activity, organ dysfunction markers and clinical observations largely capture downstream consequences of dysregulated inflammation and may therefore become abnormal only after clinically relevant injury or dysfunction has evolved. Consequently, immunologically anchored measures, potentially complemented by functional immune readouts (e.g., monocyte expression of HLA‐DR) and multi‐omics signatures, are promising candidates to guide proactive, biomarker‐driven interventions [7, 8, 12]. In addition, new facets of inflammation beyond the immune system, including the complex involvement of the endothelium in inflammation (endotheliopathy), have recently gained attention and represent promising candidates for the comprehensive analysis [36].

Biomarkers for inflammatory staging should not be judged by biological plausibility or headline diagnostic accuracy alone. Candidates should be evaluated across analytical, operational, clinical‐performance, biological‐kinetic, and stewardship‐specific dimensions (Table 2, adapted from [27, 34, 35, 37, 38, 39]).

**TABLE 2:** Dimensional framework for biomarker selection in inflammatory stewardship.

Cluster	Dimension	Key question	Stewardship implication
Analytical/pre‐analytical	Assay precision, standardization, and regulatory status	Is the biomarker measured reproducibly across time, instruments, operators, and sites? Are reference materials, harmonized protocols, or IVD‐grade assays available?	Required for serial monitoring, multicenter validation, guideline uptake, and safe interpretation of small changes
	Pre‐analytical robustness and sample burden	Is the marker stable during routine transport and processing? Does it require fresh blood, strict timing, large volumes, invasive sampling, or specialist handling?	Determines whether the marker can be used outside expert centers and repeated without excessive patient burden
Operational/health‐system	Turnaround time and workflow integration	Is the result available within the relevant decision window? Can it be integrated into routine blood draws, LIS/HIS workflows, dashboards, or ICU review cycles?	Slow or poorly integrated assays may be unsuitable for escalation or de‐escalation even when biologically informative
	Cost, reimbursement, and scalability	Is the test affordable, reimbursable, and scalable for repeated use in high‐risk perioperative or ICU populations?	Real‐world adoption depends on total clinical value, not on per‐test cost or analytical performance alone
	Guideline endorsement	Is the marker referenced in major perioperative, critical‐care, or stewardship guidelines with an explicit evidence level?	Drives institutional uptake, audit feasibility, and protocolized use
Clinical performance	Diagnostic and prognostic performance	Does the marker answer a defined clinical question with acceptable sensitivity, specificity, discrimination, and calibration? Does it also provide prognostic, predictive, or theranostic information?	Performance must be context‐specific and anchored to a defined clinical question. Discrimination, calibration, sensitivity, specificity, and predictive value should be reported separately, generic “accuracy” is insufficient for stewardship
	Cutoff and trajectory strategy	Are thresholds, gray zones, reference change values, or trajectory bands validated for the relevant population and time point?	Prevents overinterpretation of single values and supports serial, phase‐specific decision‐making
Biological/kinetic	Kinetics (onset, peak, half‐life)	Are onset, peak, half‐life, and decay compatible with the intended monitoring frequency?	Fast kinetics enable timely de‐escalation, slow kinetics are better suited for broad trend monitoring
	Pathway and compartment specificity	Does the marker reflect upstream immune activation, downstream organ injury, endothelial/thromboinflammatory activity, immunosuppression, or resolution? Is it systemic or site‐specific?	Mechanistic specificity improves endotype assignment and targeted immunomodulation, but may reduce generalizability or accessibility
	Biological variation and confounding	How strongly is the result influenced by age, frailty, comorbidity, renal/hepatic dysfunction, medication, extracorporeal support, procedure type, or baseline inflammation?	Defines the minimum interpretable change and whether individualized baselines or context‐specific interpretation are needed
Stewardship‐specific	Actionability (including de‐escalation)	Does the result trigger a predefined action: repeat staging, source evaluation, antimicrobial restraint, escalation, de‐escalation, immune attenuation, immune stimulation, or trial enrollment?	Decisive: a non‐actionable marker is operationally inferior, stewardship value is dominated by safe de‐escalation evidence
	Phenotyping capacity	Can the marker distinguish expected/resolving inflammation, excessive activation, suspected infection, immunoparalysis, or mixed states?	Enables precision immunomodulation matched to endotype, including recognition of immunoparalysis
	Theranostic linkage	Is the marker linked to a plausible intervention or safety‐monitoring strategy?	Highest clinical leverage, closes the diagnostic–therapeutic loop
	Composability and clinical utility evidence	Can the marker be combined with clinical status, organ dysfunction, and other biomarkers into an interpretable panel? Is there evidence that acting on the marker improves decisions, safety, resource use, or outcomes?	Stewardship implementation requires integrated decision support and evidence that biomarker‐guided action improves care

*Note:* Candidate biomarkers for inflammatory staging and biomarker‐guided immunomodulation should be evaluated across analytical, pre‐analytical, operational, clinical‐performance, biological‐kinetic, and stewardship‐specific dimensions. Dimensions are interdependent: Turnaround time constrains actionability, kinetics constrain monitoring frequency, and standardization gates cross‐center comparability. For inflammatory stewardship in major surgery, trauma, and related critical illness, kinetics, actionability, theranostic linkage, and clinical utility may be more decisive than headline diagnostic performance alone.

Abbreviations: HIS, hospital information system; ICU, intensive care unit; IVD, in vitro diagnostic; LIS, laboratory information system.

For bedside staging, the core clinical panel must remain low‐burden, rapidly available, and broadly deployable. High‐dimensional omics‐based assays should primarily support discovery, calibration, and compression into reduced, clinically deployable signatures before routine implementation [40]. Nonetheless, pragmatic first steps can start without the need for omics platforms: For example, (i) select one high‐risk use case with a clear time‐zero (e.g., major abdominal surgery requiring ICU admission; polytrauma with shock); (ii) define a serial core panel with actionable turnaround (vital signs/SOFA, lactate, CBC differential, CRP/PCT ± IL‐6) plus one upstream cellular readout feasible in large centers (e.g., standardized flow cytometry for monocyte HLA‐DR or neutrophil activation phenotypes); (iii) predefine deviation bands, trigger events, and conservative de‐escalation or escalation options with explicit safety stops; (iv) implement audit‐and‐feedback (exposure, infections, organ dysfunction) to iteratively refine the protocol.

For clinical implementation, inflammatory staging must compress multidimensional biology into an interpretable workflow rather than expand the number of isolated tests that clinicians must interpret in parallel. The extent of monitoring should be risk‐adapted rather than fixed. Relevant determinants include baseline frailty and comorbidity, type and magnitude of surgery or trauma, hemorrhage or shock, organ‐support requirements, chronic inflammatory or cardiovascular disease, suspected infection, extracorporeal support, and whether the patient follows an expected or deviating inflammatory trajectory [5, 23, 28]. Thus, inflammatory stewardship should begin with the question of how much immunomonitoring is justified for a given patient at a given time, not with the assumption that every patient requires the same diagnostic intensity.

In low‐ to moderate‐risk patients with an uncomplicated early postoperative or post‐traumatic course, monitoring should remain deliberately simple, low‐burden, and rapidly actionable. A minimal package may include repeated clinical assessment, vital signs, organ‐support requirements, SOFA or a comparable organ‐dysfunction score in ICU patients, lactate when hypoperfusion is suspected, complete blood count with differential, platelet count, CRP, PCT, and, where rapidly available, IL‐6 [6, 28, 34, 41, 42, 43, 44, 45] (Table 1). These markers are widely available and useful for trajectory assessment, but their interpretation is time‐dependent and context‐sensitive. CRP has delayed acute‐phase kinetics, PCT may rise after non‐infectious injury or surgery, and CRP, PCT, and IL‐6 alone are insufficient to define immune endotypes or reliably distinguish sterile postoperative or post‐traumatic inflammation from infection‐related inflammation [41, 42].

In high‐risk, deteriorating, or diagnostically ambiguous patients, a broader package may be justified to distinguish excessive inflammatory activation, suspected infection, endothelial or thromboinflammatory involvement, and immune suppression. Candidate approaches include standardized immunophenotyping, neutrophil activation markers, monocyte HLA‐DR, cytokine/chemokine profiles, and reduced molecular signatures, but these require indication‐specific validation and actionable turnaround before routine bedside use [12, 29, 30, 31, 43, 44, 45, 46].

A higher level of monitoring intensity is appropriate primarily for research, biobank‐based calibration, and adaptive interventional studies. This may include transcriptomic, proteomic, metabolomic, lipidomic, high‐dimensional cytometry, and host/pathogen sequencing approaches used to discover, validate, and reduce complex signatures into deployable clinical classifiers [29, 30, 45, 47, 48, 49]. Beyond targeted immune readouts, omics platforms offer additional opportunities for endotype discovery and risk stratification. For instance, integrating clinical variables with lipidomic, metabolomic, and proteomic data identified distinct response phenotypes after severe traumatic injury that aligned with clinical trajectories [29]. Similarly, untargeted plasma proteomics has been used to predict outcomes in patients with severe traumatic injury [30]. These approaches are not meant to move full multi‐omics into daily bedside routine. Their translational value lies in identifying reduced, standardized, and clinically interpretable signatures that can later be tested prospectively in defined use cases.

The frequency of the audit‐and‐feedback loop should also be individualized, following the general principles of prospective audit‐and‐feedback and diagnostic stewardship [27, 34]. In stable patients, reassessment may reasonably follow routine clinical review intervals, for example once daily in the ICU, where daily multidisciplinary review and an updated treatment plan are established quality indicators, or less frequently on the ward when clinical physiology is stable [9, 50, 51]. In unstable patients, reassessment should be more frequent and driven by clinical deterioration, organ‐support dynamics, new infection, reoperation, hemorrhage, ischemia–reperfusion injury, or initiation of extracorporeal support [9]. Extended immune profiling or cytokine panels should not be performed as repetitive screening without a predefined clinical question, because diagnostic stewardship emphasizes judicious, clinically contextualized testing and current immunophenotyping has not yet matured into routine unselected monitoring [27, 52, 53]. Instead, such assays should be obtained when results are likely to alter diagnostic workup, de‐escalation or escalation decisions, safety monitoring, or eligibility for biomarker‐guided trials [38, 52].

Feasibility is part of stewardship. Assays requiring excessive blood volume, specialist personnel, complex logistics, high cost, or slow turnaround will not function as bedside tools. Monitoring should be integrated with routine blood draws, use low‐volume sampling where possible, avoid non‐actionable repetition [54], and reserve labor‐intensive or blood‐volume‐intensive assays for situations in which results can change management, prognosis, or trial eligibility [55].

Beyond individual biomarkers, a usable clinical tool will require integration rather than accumulation of tests. Composite scoring frameworks, weighted multi‐marker indices, and machine‐learning‐derived trajectory classifiers may help translate complex profiles into simple, clinically interpretable categories such as “expected/resolving,” “excessive or persistent inflammatory activation,” “suspected immunosuppression,” or “mixed/uncertain” [32, 56]. These categories should be linked to predefined actions, including repeated staging, intensified diagnostic evaluation, avoidance of unnecessary immunosuppression, consideration of anti‐inflammatory or immune‐restorative strategies in defined contexts, and trial enrollment where appropriate. Data‐driven phenotyping in sepsis provides proof of concept that clinical and biomarker‐informed patterns can identify distinct phenotypes associated with host‐response differences, outcomes, and potential heterogeneity of treatment effect [32]. However, this evidence supports phenotype‐informed trial design rather than immediate bedside automation. Therefore, perioperative and trauma‐related inflammatory classifiers must be developed within defined use cases, prospectively validated, and embedded in transparent, human‐in‐the‐loop governance before routine clinical use [32, 56]. Translation should prioritize markers and panels with biological plausibility, analytical and clinical validity, clinical utility, and bedside implementability.

#### 2.2.2 Does the Patient Have the Appropriate Amount of Inflammation?

As noted above, unstratified perioperative immunomodulation has not produced consistent benefit in major surgery, as illustrated by large cardiac‐surgery corticosteroid trials [15, 16]. Accordingly, effective inflammation management hinges on identifying the patients and inflammatory endotypes/trajectories most likely to benefit and matching the therapeutic strategy to them, including timing and dose intensity.

The proposed management strategy is built on the hypothesis that, for each phase of recovery after major surgery or trauma and for each individual patient, there is a time‐dependent inflammatory target range. This target range is best defined as the level (and time course) of inflammation that is associated with favorable clinical outcomes (Figure 2), rather than a single static threshold. Following major surgery, early postoperative systemic inflammation is common and often reflects the expected host response to surgical injury. In several high‐risk surgical cohorts, including cardiac surgery, abdominal aortic aneurysm repair, and major abdominal surgery, postoperative SIRS or systemic inflammation is frequent. In these cohorts, early resolution is associated with fewer complications, whereas persistent or late SIRS is associated with infectious and non‐infectious complications, organ dysfunction, and mortality [6, 57, 58, 59]. This pattern suggests that the inflammatory target range shifts downward over time: an initially high inflammatory response may be appropriate, whereas persistently elevated inflammation later becomes maladaptive.

Mechanistic understanding supports this time dependence. Early during and after surgery, heightened inflammatory activity helps contain microbial translocation across disrupted barriers and initiates regenerative programs [7, 11]. Subsequently, inflammation should resolve to enable efficient tissue repair and remodeling [11]. It is important to note that this ideal range of inflammation is likely highly variable and influenced by both baseline and time‐varying determinants. Static factors include the patient's preexisting comorbidities [60] and genetic factors [61], while dynamic factors are dependent on the current state of disease, secondary hits (e.g., infections), and other secondary insults. These factors make the definition of a target range of inflammation highly complicated.

A central challenge is how to operationalize an individual inflammatory target range. One direct approach is to associate biomarker patterns with clinically meaningful endpoints. Although informative, this approach can lead to overly static, single‐time‐point thresholds. In practice, the target range is dynamic and should be reassessed at predefined intervals and after trigger events that change immune demand, for example, reoperation, major bleeding, ischemia–reperfusion injury, or new infection. Ideally, biomarkers should flag dysregulation, both excessive and insufficient inflammatory activity, upstream in the inflammatory cascade, when corrective action is still possible. Integration of artificial intelligence may support dynamic target‐range updates by combining multimodal inputs (biomarkers, vital signs, organ dysfunction measures, and procedural context) to generate calibrated risk estimates and decision support, ideally in a human‐in‐the‐loop model [56]. Such systems, however, require prospective validation, transparent governance, and continuous performance monitoring to ensure safety, reliability, and generalizability in intensive care practice.

In contrast, current practice relies largely on downstream signals such as organ‐dysfunction increments (e.g., ΔSOFA ≥ 2) and acute‐phase proteins with delayed kinetics, which become abnormal only after danger sensing or organ injury. Therefore, upstream markers are needed to enable timely recognition and intervention [9, 10].

#### 2.2.3 Dysregulated Acute Inflammation Can Then Be Actively Managed

Once the individualized inflammatory target range and the patient's current inflammatory status are defined, clinicians can determine whether intervention is warranted. If the status deviates from the target range (excessively or insufficiently) — a difference quantified as the inflammatory deviation — the second component, immunomodulation, applies targeted, time‐appropriate escalation or de‐escalation to restore or maintain the range. The resulting intervention space can be conceptualized along two axes: the predominant biological level of action, from upstream mechanistic targeting to downstream consequence modulation, and the dominant immune direction, from immunostimulation to immunosuppression (Figure 2).

Therapeutically, inflammatory staging should first define the intended direction of care rather than immediately select a drug. At least four broad patterns can be distinguished. First, an expected or resolving inflammatory trajectory should generally be supported and monitored rather than suppressed, with emphasis on source control when needed, adequate perfusion, oxygenation, nutrition, analgesia, early mobilization, and avoidance of unnecessary immunosuppression [8, 11, 62, 63, 64]. Second, excessive or persistent inflammatory activation with organ dysfunction should trigger intensified diagnostic reassessment and may, in selected contexts, justify attenuation or pathway‐directed modulation, provided that source control, infection, ischemia, bleeding, and other treatable drivers have been addressed [12, 13, 65, 66, 67, 68]. Third, an immune‐suppressed or immunoparalyzed trajectory, for example with lymphopenia or reduced monocyte HLA‐DR expression, should prompt heightened surveillance for secondary infection, minimization of avoidable immunosuppressive exposures, and consideration of immune‐restorative approaches in trial settings [69, 70, 71, 72]. Fourth, mixed or uncertain trajectories should lead to repeated staging after clinically relevant trigger events, such as reoperation, ischemia–reperfusion injury, hemorrhage, new infection, or initiation of extracorporeal support. Thus, the immediate output of inflammatory staging should be a conservative therapeutic orientation rather than an isolated biomarker‐triggered intervention.

A diverse repertoire of direct and indirect immunomodulatory strategies is under evaluation for perioperative and trauma‐related inflammation, but most are not established as routine care in these settings (Figure 2). Systemic corticosteroids have not shown sufficient overall benefit in large unselected cardiac‐surgery trials to support routine use without biomarker‐based stratification [15, 16]. Targeted cytokine or pathway inhibitors, extracorporeal mediator‐removal approaches (with overall neutral trial results in unselected populations), therapeutic plasma exchange, plasma‐derived interventions, mesenchymal stromal cells, and immune‐restorative strategies may be biologically plausible in selected inflammatory states, but perioperative and trauma applications require biomarker‐defined endotypes, safety monitoring, and prospective validation (Figure 2, [65, 66, 67, 68, 71, 72, 73, 74]).

Cardiovascular inflammation provides a useful proof of principle but not a direct perioperative indication. Low‐dose colchicine reduced ischemic events in selected trials of chronic coronary disease and post‐myocardial‐infarction secondary prevention, whereas a recent large acute‐MI trial was neutral for its primary clinical endpoint [75, 76, 77]. Contemporary coronary disease guidelines consider low‐dose colchicine in selected patients [78, 79]. IL‐1β inhibition has shown that targeted anti‐inflammatory therapy can reduce vascular events in patients with residual inflammatory risk [20], and NLRP3 biology provides a mechanistic rationale for this pathway [80]. These observations support the biological plausibility of pathway‐directed immunomodulation, but extrapolation to acute perioperative or post‐traumatic inflammation should remain trial‐based.

Cellular therapies (CAR‐T, mesenchymal stromal cells, regulatory T‐cell products) represent an emerging frontier. For example, mesenchymal stromal cells show immunomodulatory effects and acceptable safety in ARDS trials [74], but efficacy is inconsistent and applications remain investigational in perioperative/trauma care.

Of note, inflammatory stewardship should include not only direct immunomodulatory interventions, but also indirect measures that influence the inflammatory load. In the operative setting, surgical strategy matters: minimizing operative time and tissue trauma, applying Enhanced Recovery After Surgery (ERAS) principles and prehabilitation approaches, and making context‐specific choices can blunt the perioperative inflammatory response [64, 81]. In cardiac surgery, choosing off‐pump over on‐pump CABG can attenuate cytokine surges and systemic inflammation, although consistent clinical outcome benefits have not been demonstrated [82]. In trauma, deciding between Early Total Care (ETC) and Damage‐Control Orthopedics/Surgery (DCO/DCS) is a key lever to avoid or reduce a harmful ‘second hit’ [83].

Finally, active inflammation management must also include the option to stimulate the inflammatory response. After major surgery and trauma, many patients develop features of postoperative immunosuppression (e.g., lymphopenia, reduced expression of HLA‐DR on monocytes), which are associated with secondary infections and worse outcomes [69, 70]. Evidence from critical‐illness trials shows that immunostimulatory therapies can reverse key immune defects. For example, GM‐CSF can enhance neutrophil function and interleukin‐7 reverses sepsis‐associated lymphopenia [71, 72]. However, patient‐centered outcome benefits remain unproven, and these approaches are not routine in perioperative/trauma care. Taken together, a comprehensive inflammatory management strategy must also be able to stimulate the inflammatory response, if necessary.

## 3 Summary and Outlook

In summary, inflammatory stewardship provides a structured precision‐medicine framework for staging and actively managing acute inflammation after major surgery and trauma. Its central premise is that inflammatory trajectories should be interpreted against phase‐specific, patient‐adapted target ranges and linked to conservative, evidence‐guided escalation or de‐escalation when the trajectory deviates (Figures 1 and 2).

Clinical implementation will require large, stratified evaluations across heterogeneous surgical and trauma populations. Research methods such as high‐dimensional immunophenotyping, host‐response profiling, third‐generation sequencing, and emerging protein‐detection technologies should be translated into standardized, low‐burden, clinically interpretable assays before routine deployment.

Achieving inflammatory stewardship will require advances in biomarker development, standardization, and deeper mechanistic insight into immune dysregulation. If successful, this framework could shift care from reacting to inflammation after organ injury toward proactive, trajectory‐based control supported by real‐time immunomonitoring. The goal is to prevent maladaptive inflammatory states, preserve immune homeostasis, and improve outcomes. Over time, inflammatory stewardship could evolve into a dedicated clinical competency integrating perioperative and critical care disciplines with standardized diagnostics, protocols, and governance structures.

## Author Contributions

Writing – original draft: L.F.H. and D.A.C.M. Writing – review and editing: All authors.

## Funding

This work was supported by the German Research Foundation (project numbers 545061513 and 568940582) and the Else Kröner‐Fresenius Foundation (Else Kröner‐Fresenius‐Stiftung, project number 2021_EKEA.112).

## Ethics Statement

The authors have nothing to report.

## Consent

The authors have nothing to report.

## Conflicts of Interest

The authors declare no conflicts of interest.

## Data Availability

The authors have nothing to report.
